# Face Perception and Test Reliabilities in Congenital Prosopagnosia in Seven Tests

**DOI:** 10.1177/2041669515625797

**Published:** 2016-01-20

**Authors:** Janina Esins, Johannes Schultz, Claudia Stemper, Ingo Kennerknecht, Isabelle Bülthoff

**Affiliations:** Department of Human Perception, Cognition and Action, Max Planck Institute for Biological Cybernetics, Tübingen, Germany; Department of Psychology, Durham University, Durham, UK; Institute of Human Genetics, Westfälische Wilhelms-Universität Münster, Münster, Germany; Institute of Human Genetics, Westfälische Wilhelms-Universität Münster, Münster, Germany; Department of Human Perception, Cognition and Action, Max Planck Institute for Biological Cybernetics, Tübingen, Germany

**Keywords:** Congenital prosopagnosia, developmental prosopagnosia, face recognition, test reliability, Cronbach’s alpha

## Abstract

Congenital prosopagnosia, the innate impairment in recognizing faces, is a very heterogeneous disorder with different phenotypical manifestations. To investigate the nature of prosopagnosia in more detail, we tested 16 prosopagnosics and 21 controls with an extended test battery addressing various aspects of face recognition. Our results show that prosopagnosics exhibited significant impairments in several face recognition tasks: impaired holistic processing (they were tested amongst others with the Cambridge Face Memory Test (CFMT)) as well as reduced processing of configural information of faces. This test battery also revealed some new findings. While controls recognized moving faces better than static faces, prosopagnosics did not exhibit this effect. Furthermore, prosopagnosics had significantly impaired gender recognition—which is shown on a groupwise level for the first time in our study. There was no difference between groups in the automatic extraction of face identity information or in object recognition as tested with the Cambridge Car Memory Test. In addition, a methodological analysis of the tests revealed reduced reliability for holistic face processing tests in prosopagnosics. To our knowledge, this is the first study to show that prosopagnosics showed a significantly reduced reliability coefficient (Cronbach’s alpha) in the CFMT compared to the controls. We suggest that compensatory strategies employed by the prosopagnosics might be the cause for the vast variety of response patterns revealed by the reduced test reliability. This finding raises the question whether classical face tests measure the same perceptual processes in controls and prosopagnosics.

## Introduction

Congenital prosopagnosia refers to the lifelong, innate impairment in identifying someone by his or her face (first case description by McConachie, 1976). It is estimated to affect about 2% of the population (Bowles et al., 2009; Kennerknecht, Grüter, Welling, & Wentzek, 2006; Kennerknecht, Ho, & Wong, 2008) and is characterized as a neurodevelopmental disorder of face recognition without any deficits in low-level vision or intelligence (Behrmann & Avidan, 2005). Face perception is an increasing subject of interest for research, and investigating prosopagnosia is one way of gaining a better understanding of how the human recognition systems works.

Two main aspects of face perception put faces apart from most other objects: (1). Faces are recognized at the individual level (identification); (2). They are processed holistically. While identification is a clear concept, what exactly is meant with the term “holistic processing” is not well defined and there are numerous controversies about the use of the terms holistic and configural processing (e.g., McKone & Yovel, 2009; Maurer, Le Grand, & Mondloch, 2002; Piepers & Robbins, 2012; Rossion, 2013). Here we used these terms following the definitions given by Maurer, Le Grand, and Mondloch (2002, p. 255): Holistic processing is defined as a perceptual phenomenon “glueing together the features into a gestalt” and the concept configural processing refers to “processing second-order relations (i.e., the spacing among features).” We also use the concept of featural processing to refer to processing the features of the face (e.g., the shape, color and texture of the eyes, mouth, nose, etc.). Finally, we view holistic processing as relying at least in part on configural and featural processing.

Different experimental approaches exist to measure holistic processing, for example, the *part-whole test* (Tanaka & Farah, 1993), *the composite face test* (Young, Hellawell, & Hay, 1987), or the manipulation of configural and featural information of faces (Le Grand et al., 2006; Yovel & Duchaine, 2006). The extent to which these approaches measure the “same” holistic processes was examined by several studies applying different holistic face recognition tests to the same participants. While DeGutis and colleagues were able to find a significant correlation between the *part-whole test* and the *composite face test* (Degutis, Wilmer, Mercado, & Cohan, 2013), a study by Wang and colleagues did not find such a correlation (Wang, Li, Fang, Tian, & Liu, 2012). Therefore, the question whether the tests tap into the same holistic mechanisms is yet to be answered. However, in both studies the performance in either test was significantly correlated to face recognition performance, confirming previous findings of a correlation between holistic processing and face individuation (Richler, Cheung, & Gauthier, 2011).

Not only face identification but also holistic, configural and featural processing are believed to be impaired in congenital prosopagnosia. However, controversy reigns as psychophysical studies differ in their findings. While several studies found evidence for weaker holistic processing (Avidan, Tanzer, & Behrmann, 2011; Palermo et al., 2011), other studies reported that only one of their respective prosopagnosic participants showed reduced holistic processing (Le Grand et al., 2006; Rivolta, Palermo, Schmalzl, & Williams, 2012). Similarly, evidence of reduced configural or featural sensitivity varies depending on the studies (see Lobmaier, Bölte, Mast, & Dobel, 2010 and Yovel & Duchaine, 2006 for evidence of an impairment and Le Grand et al., 2006 for contradictory findings). Other deficits of face processing in developmental prosopagnosia are also subject of debate. For example, some studies found impaired gender recognition in congenital prosopagnosics (Ariel & Sadeh, 1996; Duchaine & Nakayama, 2006a), while others reported gender recognition to be normal (Chatterjee & Nakayama, 2012). Also, some, but not all prosopagnosic participants show impairments in object recognition (Kress & Daum, 2003; Le Grand et al., 2006).

In short, the picture of a very heterogeneous disorder, even across prosopagnosics belonging to the same family, emerges from these results (Le Grand et al., 2006; Lee et al., 2010; Schmalzl, Palermo, & Coltheart, 2008; Schweich & Bruyer, 1993). This heterogeneity is evident even when accounting for differences in experiment and stimulus design and needs clarification. Further, a better characterization of prosopagnosia might help gain a better understanding of face processing. For these reasons, we tested face perception in congenital prosopagnosia in more details. We developed new tests assessing so far untested aspects of face perception (e.g., the influence of strategy usage on test results) as well as aspects for which controversial results exist in literature (e.g., gender recognition). In addition, we included two widely used tests for reference, the Cambridge Face Memory test (CFMT, Duchaine and Nakayama, 2006b) and the Cambridge Car Memory Test (CCMT, Dennett et al., 2011).

This paper contains two main parts. The first is a detailed performance analysis of prosopagnosic and control participants on several psychophysical tests, allowing to deepen the understanding of the heterogeneous appearance of prosopagnosia. We report and compare the performance of a group of 16 congenital prosopagnosics to the performance of 21 matched controls in seven tests. Our tests aimed at measuring holistic face processing, configural and featural face processing, processing of faces in motion, strategy usage when recognizing faces, face gender recognition, and object recognition. For each test separately, we will present motivation, methodological details, results, and discussion. The second part examines test reliability. To verify the quality of our newly created tests, we calculated their reliabilities and compared reliabilities values of old and new tests across participant groups. Those data are discussed in view of participants’ performance for the tests presented in the first part. The paper ends by a general discussion of our findings and their implications.

## General Methods

### Procedure

The experiments were conducted in two sessions lying about 2 years apart: On average, 24.6 months (*SD* = 2.3) for prosopagnosics and 20.3 months (*SD* = 1.6) for controls. During the first session, participants performed the CFMT, test number 1, a surprise recognition test (number 3), and a similarity rating test (5). The second session included the CCMT, 2, the composite face test (4), a gender recognition test (6), and a facial motion advantage test (7). In both sessions, participants could take self-paced breaks between the experiments.

All participants were tested individually. The experiments were run on a desktop PC with 24″ screen. The CFMT and CCMT are Java-script based; the other experiments were run with Matlab2011b (The MathWorks Inc., n.d.) and Psychtoolbox (Brainard, 1997; Kleiner, Brainard, & Pelli, 2007). Participants were seated at a viewing distance of approximately 60 cm from the screen. The procedure was approved by the local ethics committee.

### Participants

We tested 16 congenital prosopagnosic participants (from now on referred to as “prosopagnosics”) and 21 control participants (“controls”) matched as closely as possible to the prosopagnosic participants in terms of age and sex (see Table 1).

**Table 1.** Participants’ Demographics.

	Prosopagnosics		Controls	
	Sex	Age	Sex	Age
1	f	22	f	21
2	f	24	f	24
3	f	27	f	24
4	f	28	f	28
5	m	33	f	29
6	m	34	f	31
7	f	36	m	33
8	m	36	m	36
9	m	37	m	37
10	f	41	f	37
11	f	46	m	38
12	m	47	m	39
13	m	52	m	39
14	f	54	f	42
15	m	57	m	44
16	m	59	f	44
17			f	47
18			m	48
19			f	49
20			f	58
21			m	60
♂	8		9	
Mean age		39.6		38.5

Sex (“m” = male, “f” = female) and age (in years) of prosopagnosic and control participants

All participants provided informed consent. All participants had normal or corrected-to-normal visual acuity, but we did not formally assess color perception, contrast sensitivity, stereoscopic vision or other neuropsychological measures or personality traits (e.g., autism). As no brain imaging was available to exclude the presence of brain lesions, we relied on participants’ self-testimonies or parents’ testimonies. To provide an objective measure of face processing abilities and to maintain comparability with other studies, we tested all participants with the CFMT. Individual results and z-scores are given in Tables 2 (raw scores) and 3 (z-scores) for prosopagnosics and Tables 4 (raw scores) and 5 (z-scores) for controls. Tables 2 to 5 also contain the raw scores and z-scores of all other tests reported in this study. Z-scores for both groups were calculated based on the results of the control participants.

**Table 2.** Prosopagnosics’ Behavioral Data: Raw Scores.

	3.1. CFMT	3.2. Cars		3.3. Surprise recognition		3.4. Composite face task				3.5. Featural and configural sensitivity task		3.6. Gender	3.7. Facial motion advantage			
		CCMT	Car expert.	Surprise	Control	Up- algn	Up- misalgn	Inv- algn	Inv- misalgn	Features	Configuration		id-dyn	id-stat	ch-dyn	ch-stat
1	0.63	0.96	0.69	2.19	0.90	0.71	0.49	−0.72	−0.41	1.08	0.57	0.77	2.44	2.19	1.36	1.11
2	0.50	0.60	0.31	0.63	1.40	0.39	0.24	−0.27	0.56	1.32	0.93	0.77	2.36	2.02	0.62	2.02
3	0.51	0.61	0.25	0.69	1.59	−0.49	0.41	−0.55	−0.19	0.93	0.83	0.80	2.35	1.15	0.32	1.15
4	0.60	0.97	0.88	0.18	2.18	−0.63	−0.46	0.29	−0.72	0.93	0.43	0.77	1.20	1.99	−0.79	1.20
5	0.64	0.79	0.31	1.05	−0.14	1.04	0.00	−0.05	0.34	0.96	0.79	0.91	0.57	1.40	0.90	1.73
6	0.53	0.69	0.38	0.48	0.87	0.74	0.80	0.62	0.51	1.05	0.64	0.85	1.20	1.11	0.90	0.54
7	0.46	0.54	0.50	0.87	0.90	0.68	−0.75	−0.10	0.15	1.04	0.63	0.89	1.50	0.29	1.05	0.62
8	0.51	0.75	0.44	1.31	1.31	1.87	0.66	−0.44	0.44	0.88	0.29	0.95	1.99	1.73	0.90	0.54
9	0.54	0.82	0.69	1.08	1.81	0.39	0.61	−0.31	0.71	1.57	0.10	0.86	1.22	1.81	0.43	1.02
10	0.56	0.68	0.56	0.59	2.81	0.72	0.29	0.33	0.61	1.24	0.55	0.91	3.19	2.36	2.36	1.73
11	0.47	0.90	0.69	1.50	1.65	1.14	0.54	−0.80	0.22	1.12	0.53	0.82	2.81	2.56	1.65	0.83
12	0.53	0.75	0.31	0.73	0.00	1.31	0.77	−0.66	0.67	1.12	0.34	0.81	1.45	2.44	1.73	1.08
13	0.63	0.71	0.50	0.54	1.36	0.40	0.30	−0.14	−0.28	0.91	0.26	0.77	1.16	1.73	0.83	1.16
14	0.47	0.85	0.88	1.08	1.11	1.04	0.33	0.10	0.55	0.84	0.17	0.86	1.73	2.44	1.73	1.36
15	0.61	0.64	0.38	0.59	1.02	0.57	−0.30	0.63	0.84	1.39	0.53	0.91	1.99	1.99	1.99	0.90
16	0.58	0.75	0.56	0.14	1.20	0.44	1.05	0.23	0.30	1.05	0.59	0.81	2.81	2.36	1.45	2.02
Mean	0.55	0.75	0.52	0.85	1.25	0.65	0.31	−0.11	0.27	1.09	0.51	0.84	1.87	1.85	1.09	1.19
*SD*	0.06	0.13	0.20	0.52	0.73	0.61	0.48	0.46	0.45	0.20	0.23	0.06	0.74	0.61	0.76	0.48

CFMT = Cambridge Face Memory Test; CCMT = Cambridge Car Memory Test; expert. = expertise; up = upright; inv = inverted; algn = aligned; mislagn = misaligned; id = identical; ch = changed; dyn = dynamic; stat = static. Raw scores are given in percent correct for CFMT, CCMT, and gender recognition. All other scores depict *d*’.

**Table 3.** Prosopagnosics’ Behavioral Data: Z-Scores.

	3.1. CFMT	3.2. Cars		3.3. Surprise recognition		3.4. Composite face task				3.5. Featural and configural sensitivity task		3.6. Gender	3.7. Facial motion advantage			
		CCMT	Car expert.	Surprise	Control	Up- algn	Up- misalgn	Inv- algn	Inv- misalgn	Features	Configuration		id-dyn	id-stat	ch-dyn	ch-stat
1	−1.96*	1.43	0.26	0.68	−1.77	−0.49	0.48	−1.55	−1.06	−0.66	−0.86	−2.93*	−0.65	−0.10	−0.51	−0.41
2	−3.28*	−1.38	−2.07*	−1.39	−1.12	−0.74	0.17	−0.90	0.83	0.41	1.50	−2.93*	−0.81	−0.35	−1.55	0.89
3	−3.14*	−1.27	−2.46*	−1.42	−0.87	−1.43	0.39	−1.31	−0.64	−1.29	0.81	−2.41*	−0.84	−1.71	−1.97*	−0.35
4	−2.26*	1.54	1.42	−2.12*	−0.13	−1.54	−0.70	−0.09	−1.66	−1.29	−1.75	−2.93*	−3.01*	−0.41	−3.54*	−0.28
5	−1.81	0.13	−2.07*	−1.00	−3.09*	−0.23	−0.13	−0.59	0.40	−1.16	0.57	−0.05	−4.19*	−1.32	−1.15	0.47
6	−2.99*	−0.62	−1.68	−1.72	−1.82	−0.46	0.88	0.38	0.72	−0.77	−0.41	−1.36	−3.01*	−1.77	−1.15	−1.21
7	−3.73*	−1.81	−0.91	−1.24	−1.77	−0.51	−1.07	−0.65	0.02	−0.80	−0.49	−0.57	−2.43*	−3.04*	−0.95	−1.08
8	−3.14*	−0.19	−1.29	−0.61	−1.22	0.43	0.70	−1.15	0.59	−1.54	−2.65*	0.74	−1.52	−0.81	−1.15	−1.21
9	−2.84*	0.35	0.26	−0.80	−0.49	−0.74	0.64	−0.97	1.10	1.51	−3.91*	−1.10	−2.96*	−0.68	−1.82	−0.53
10	−2.70*	−0.73	−0.52	−1.60	0.81	−0.48	0.24	−0.04	0.92	0.05	−0.98	−0.05	0.75	0.16	0.90	0.48
11	−3.58*	1.00	0.26	−0.26	−0.70	−0.15	0.55	−1.66	0.16	−0.47	−1.14	−1.88	0.05	0.48	−0.10	−0.80
12	−2.99*	−0.19	−2.07*	−1.42	−2.91*	−0.01	0.84	−1.46	1.02	−0.47	−2.32*	−2.15*	−2.52*	0.29	0.02	−0.44
13	−1.96*	−0.51	−0.91	−1.61	−1.06	−0.73	0.25	−0.72	−0.81	−1.37	−2.89*	−2.93*	−3.07*	−0.80	−1.26	−0.33
14	−3.58*	0.56	1.42	−0.80	−1.48	−0.22	0.29	−0.37	0.81	−1.70	−3.46*	−1.10	−1.99*	0.29	0.02	−0.05
15	−2.11*	−1.05	−1.68	−1.60	−1.64	−0.60	−0.50	0.39	1.36	0.71	−1.10	−0.05	−1.52	−0.41	0.37	−0.69
16	−2.40*	−0.19	−0.52	−2.18*	−1.41	−0.70	1.19	−0.18	0.31	−0.77	−0.74	−2.15*	0.05	0.16	−0.38	0.89
Mean	−2.78	−0.18	−0.79	−1.19	−1.29	−0.54	0.26	−0.68	0.25	−0.60	−1.24	−1.49	−1.73	−0.63	−0.89	−0.29
*SD*	0.63	0.98	1.23	0.72	0.96	0.48	0.61	0.66	0.87	0.88	1.53	1.23	1.40	0.95	1.07	0.68

CFMT = Cambridge Face Memory Test; CCMT = Cambridge Car Memory Test; expert. = expertise; up = upright; inv = inverted; algn = aligned; mislagn = misaligned; id = identical; ch = changed; dyn = dynamic; stat = static. Z-scores were calculated for each tests based on mean and standard deviation of the corresponding performance of the 21 control participants in this study.

* Z-scores of less than −1.96.

**Table 4.** Controls’ Behavioral Data: Raw Scores.

	3.1. CFMT	3.2. Cars		3.3. Surprise recognition		3.4. Composite face task				3.5. Featural and configural sensitivity task		3.6. Gender	3.7. Facial motion advantage			
		CCMT	Car expert.	Surprise	Control	Up- algn	Up- misalgn	Inv- algn	Inv- misalgn	Features	Configuration		id-dyn	id-stat	ch-dyn	ch-stat
1	0.85	0.93	0.50	1.56	2.44	1.31	0.31	0.77	−0.17	1.36	0.74	0.88	2.81	2.36	1.99	0.90
2	0.81	0.86	0.63	1.35	1.81	4.81	0.27	0.63	−0.66	1.04	0.67	0.80	2.18	0.62	1.81	−0.28
3	0.99	0.93	0.94	2.81	3.19	2.32	0.00	0.85	0.00	1.36	0.86	0.86	1.65	2.81	1.65	2.81
4	0.89	0.71	0.50	3.19	3.19	1.40	0.53	0.00	0.44	1.04	0.79	0.88	2.44	1.35	1.65	1.02
5	0.96	0.86	0.94	1.81	1.73	3.08	0.41	0.14	0.53	0.98	0.70	0.91	3.19	2.81	2.36	1.36
6	0.85	0.81	0.56	2.44	1.20	1.24	0.27	1.16	0.22	1.39	0.60	0.93	3.51	2.81	1.48	1.08
7	0.83	0.56	0.63	1.20	0.87	0.06	−0.46	0.09	0.17	1.49	0.97	0.94	2.56	1.73	1.11	0.83
8	0.96	0.78	0.50	2.56	3.19	0.29	−1.16	0.99	0.55	1.31	0.69	0.89	3.19	1.73	2.81	2.19
9	0.67	0.75	0.63	0.97	1.65	−0.55	−0.14	2.16	0.24	1.23	0.88	0.94	3.51	1.40	1.77	1.73
10	0.72	0.64	0.75	0.87	2.81	0.88	1.57	0.87	0.00	1.25	0.53	0.96	2.81	1.99	1.65	0.79
11	0.67	0.56	0.56	0.57	3.19	1.11	0.06	0.22	0.55	1.57	0.86	0.89	2.81	2.44	1.36	1.99
12	0.78	0.51	0.25	1.99	2.02	1.28	0.31	−1.16	0.24	1.13	0.66	0.95	2.81	3.19	1.99	1.16
13	0.69	0.81	0.75	1.81	1.20	0.82	0.11	0.00	1.16	0.84	0.57	0.94	2.36	1.73	0.83	1.45
14	0.75	0.67	0.50	1.99	2.44	1.51	−0.85	−0.85	0.04	1.52	0.72	0.96	3.19	2.44	2.36	1.08
15	0.86	0.93	0.75	1.88	2.02	0.46	−1.07	0.39	−0.22	0.86	0.64	0.95	3.51	2.18	3.51	1.02
16	0.72	0.83	0.63	1.81	2.19	3.57	2.15	0.27	0.59	1.40	0.96	0.94	1.99	2.44	1.08	1.36
17	0.81	0.76	0.88	0.59	1.99	1.11	0.00	0.26	−1.16	1.17	0.71	0.93	2.81	2.81	1.45	1.73
18	0.74	0.83	0.63	1.02	3.19	0.27	−1.14	0.22	−0.17	1.30	0.48	0.99	3.19	2.81	1.16	1.36
19	0.85	0.88	0.69	2.56	3.19	0.03	0.55	0.31	−0.55	1.04	0.38	0.91	2.02	2.81	0.29	2.81
20	0.89	0.93	0.69	2.19	2.36	1.86	0.29	−0.33	0.55	1.54	0.76	0.96	3.19	2.81	1.45	1.99
21	0.75	0.74	0.69	1.50	1.08	0.98	0.06	0.55	0.51	0.91	0.56	0.82	2.81	1.99	2.36	0.90
Mean	0.81	0.77	0.65	1.75	2.23	1.33	0.10	0.36	0.14	1.23	0.70	0.91	2.79	2.25	1.72	1.40
*SD*	0.09	0.13	0.16	0.73	0.78	1.27	0.80	0.70	0.52	0.23	0.15	0.05	0.53	0.65	0.71	0.71

CFMT = Cambridge Face Memory Test; CCMT = Cambridge Car Memory Test; expert = expertise; up = upright; inv = inverted; algn = aligned; mislagn = misaligned; id = identical; ch = changed; dyn = dynamic; stat = static. Raw scores are given in percent correct for CFMT, CCMT, and gender recognition. All other scores depict d.

**Table 5.** Controls’ Behavioral Data: z-Scores.

	3.1. CFMT	3.2. Cars		3.3. Surprise recognition		3.4. Composite face task				3.5. Featural and configural sensitivity task		3.6. Gender	3.7. Facial motion advantage			
		CCMT	Car expert.	Surprise	Control	Up-algn	Up- misalgn	Inv- algn	Inv- misalgn	Features	Configuration		id-dyn	id-stat	ch-dyn	ch-stat
1	0.39	1.21	−0.91	−0.29	0.45	−0.01	0.27	0.59	−0.59	0.58	0.28	−0.84	0.05	0.16	0.37	−0.69
2	−0.05	0.67	−0.13	−0.59	−0.49	2.75	0.21	0.39	−1.54	−0.80	−0.21	−2.41*	−1.14	−2.52*	0.13	−2.36*
3	1.86	1.21	1.81	1.48	1.17	0.79	−0.13	0.70	−0.26	0.58	1.05	−1.10	−2.15*	0.87	−0.10	2.00
4	0.83	−0.51	−0.91	1.86	1.17	0.06	0.54	−0.52	0.58	−0.83	0.61	−0.84	−0.65	−1.39	−0.10	−0.53
5	1.57	0.67	1.81	0.14	−0.68	1.39	0.39	−0.31	0.77	−1.07	0.00	−0.05	0.75	0.87	0.90	−0.05
6	0.39	0.24	−0.52	1.11	−1.41	−0.07	0.21	1.15	0.16	0.71	−0.65	0.21	1.36	0.87	−0.33	−0.44
7	0.25	−1.70	−0.13	−0.80	−1.82	−1.00	−0.69	−0.38	0.06	1.18	1.74	0.47	−0.43	−0.81	−0.87	−0.80
8	1.57	0.03	−0.91	1.05	1.17	−0.82	−1.58	0.90	0.80	0.38	−0.09	−0.57	0.75	−0.81	1.54	1.12
9	−1.52	−0.19	−0.13	−1.06	−0.70	−1.48	−0.30	2.58	0.21	0.00	1.13	0.47	1.36	−1.32	0.08	0.47
10	−0.93	−1.05	0.65	−1.24	0.81	−0.35	1.84	0.73	−0.26	0.11	−1.14	1.00	0.05	−0.41	−0.10	−0.85
11	−1.52	−1.70	−0.52	−1.63	1.17	−0.17	−0.05	−0.20	0.81	1.51	1.01	−0.57	0.05	0.29	−0.51	0.83
12	−0.34	−2.02*	−2.46*	0.38	−0.34	−0.03	0.27	−2.18*	0.20	−0.41	−0.29	0.74	0.05	1.45	0.37	−0.33
13	−1.23	0.24	0.65	0.14	−1.41	−0.40	0.02	−0.52	1.98	−1.70	−0.86	0.47	−0.81	−0.80	−1.26	0.08
14	−0.64	−0.84	−0.91	0.38	0.45	0.14	−1.18	−1.73	−0.19	1.29	0.12	1.00	0.75	0.29	0.90	−0.44
15	0.54	1.21	0.65	0.11	−0.34	−0.68	−1.46	0.04	−0.69	−1.59	−0.37	0.74	1.36	−0.11	2.52	−0.53
16	−0.93	0.46	−0.13	0.14	0.03	1.78	2.56	−0.13	0.87	0.77	1.66	0.47	−1.52	0.29	−0.90	−0.05
17	−0.05	−0.08	1.42	−1.60	−0.26	−0.17	−0.13	−0.14	−2.51*	−0.25	0.08	0.21	0.05	0.87	−0.38	0.48
18	−0.78	0.46	−0.13	−1.04	1.17	−0.83	−1.54	−0.20	−0.59	0.33	−1.43	1.52	0.75	0.87	−0.79	−0.05
19	0.39	0.78	0.26	1.05	1.17	−1.03	0.56	−0.07	−1.33	−0.80	−2.12*	−0.05	−1.44	0.87	−2.02*	2.00
20	0.83	1.21	0.26	0.68	0.10	0.43	0.24	−0.99	0.80	1.40	0.40	1.00	0.75	0.87	−0.38	0.83
21	−0.64	−0.30	0.26	−0.26	−1.40	−0.28	−0.05	0.28	0.73	−1.37	−0.94	−1.88	0.05	−0.41	0.90	−0.69
Mean	0.00	0.00	0.00	0.00	0.00	0.00	0.00	0.00	0.00	0.00	0.00	0.00	0.00	0.00	0.00	0.00
*SD*	1.00	1.00	1.00	1.00	1.00	1.00	1.00	1.00	1.00	1.00	1.00	1.00	1.00	1.00	1.00	1.00

CFMT = Cambridge Face Memory Test; CCMT = Cambridge Car Memory Test; expert. = expertise; up = upright; inv = inverted; algn = aligned; mislagn = misaligned; id = identical; ch = changed; dyn = dynamic; stat = static. Z-scores were calculated for each tests based on mean and standard deviation of the corresponding performance of the 21 control participants in this study.

* Z-scores of less than −1.96.

#### Prosopagnosics

The prosopagnosics were diagnosed by a semi-structured interview (Kennerknecht et al., 2008; Kennerknecht, Plümpe, Edwards, & Raman, 2007; Stollhoff, Jost, Elze, & Kennerknecht, 2011), approved by the ethical committee of the University of Münster, Germany, protocol No 3XKenn2. All prosopagnosics were compensated with 8 Euro per hour plus travel expenses.

#### Controls

All controls were compensated with 8 Euro per hour. The controls did not participate in the full diagnostic interview but in a questionnaire and reported to have no problems in recognizing faces of their friends and family members.

### Analysis

The description of the dependent variables is given for each test individually. All analyses were conducted with Matlab2011b (The MathWorks Inc.) and IBM SPSS statistics Version 20 (IBM Corp. Released 2011). Analysis of variances (ANOVAs) and their effect sizes (*η^2^*) and linear regressions were calculated with IBM SPSS statistics Version 20. *T* tests and their effect sizes Cohen’s *d* (*d*), Mann–Whitney *U* tests and tests’ internal consistency reliability coefficients were calculated with Matlab2011b.

Where possible, tests reliability was calculated as Cronbach’s alpha with the function cronbach.m for Matlab (Leontitsis, 2005). Furthermore, we calculated reliability with the split-half method and subsequent adjustment with the Spearman–Brown prediction formula for all tests: The trials of a test are split into halves (e.g., first half versus second half, or odd trials versus even trials). Then the mean score of each half is calculated for each participant. The correlation between participant’s mean half scores gives an estimate of the test reliability (Davidshofer & Murphy, 2005). We adapted this method by bootstrapping: Test trials were split randomly into halves, followed by correlation of the mean half scores. This procedure was repeated 100,000 times. The median of these bootstrapped correlations was then adjusted to the tests full length with the Spearman–Brown prediction formula (Brown, 1910; Spearman, 1910). Statistical difference between prosopagnosics’ and controls’ reliability coefficients for Cronbach’s alpha was calculated based on the Fisher–Bonett approach (Bonett, 2003;^1^ Formula (2)). Statistical difference between prosopagnosics’ and controls’ split-half reliability coefficients was calculated as statistical difference between correlation coefficients (Fisher, 1921). This was done for the uncorrected reliability coefficients (i.e., before applying the Spearman–Brown prediction formula).

Reaction times of correctly answered trials were also analyzed. For space reasons, we do not report them as they confirm all accuracy data and therefore do not add any additional information.

## Tests

### CFMT

#### Motivation

The CFMT was created and provided by Duchaine and Nakayama (2006b). It is a widely used test to characterize prosopagnosics (Kimchi, Behrmann, Avidan, & Amishav, 2012; Rivolta, Palermo, Schmalzl, & Coltheart, 2011) and to assess holistic face recognition abilities. The CFMT has been confirmed to have a high internal consistency reliability with a Cronbach’s alpha between .8 and .9 in different studies (Bowles et al., 2009; Herzmann, Danthiir, Schacht, Sommer, & Wilhelm, 2008; Wilmer et al., 2010). We used this test as an objective measure of face recognition abilities of our participants, expecting reduced recognition abilities for the prosopagnosic group, and to allow comparison with other studies.

#### Stimuli and task

As this test has been described in detail in the original study (Duchaine & Nakayama, 2006b), only a short description is given here. Portraits of male Caucasians serve as stimuli. The participants were familiarized with six target faces, which they then had to recognize among distractor faces in a three-alternative-forced-choice task. Difficulty was increased stepwise during the test by changing viewpoints and lighting conditions and adding noise. Participants had to decide for each image whether the face had been seen before or not by pressing the relevant keys on the keyboard. The next image appeared as soon as an answer was entered. No feedback was given and no time restrictions were applied. The test can be run in an upright and inverted condition. We only used the upright condition. In our setting, the stimuli faces had a visual angle of 5.7° horizontally and vertically.

#### Results

**Figure 1.**
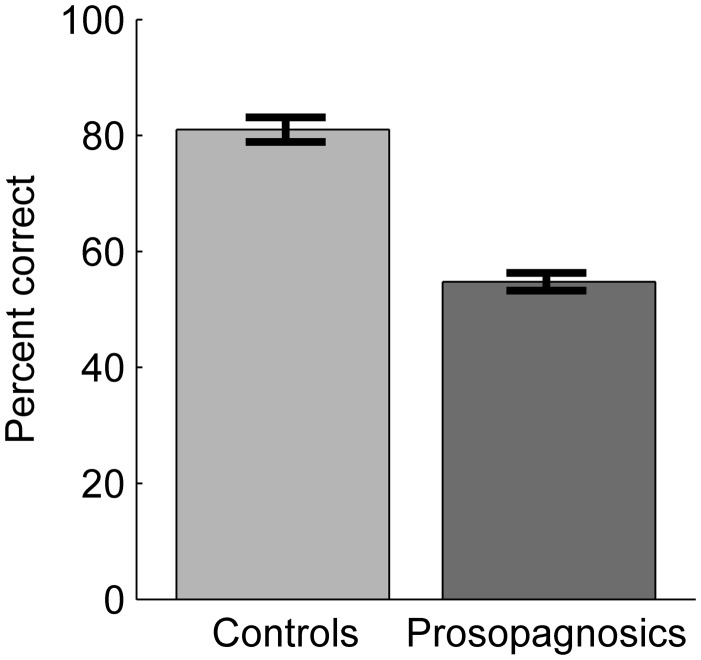
Mean percentage of correctly recognized faces in the CFMT for controls and prosopagnosics. Error bars: *SEM*. CFMT = Cambridge Face Memory Test.

We calculated the overall recognition performance as the percentage of correctly recognized faces per participant. Figure 1 depicts the mean scores per group. Controls correctly recognized 81.0% (*SD* = 9.4) of the test faces, while prosopagnosics scored 54.8%, (*SD* = 5.9). The difference between groups was significant (one-way ANOVA: *F*(1, 36) = 94.7, *p* < .001, *η^2^* = .73), with prosopagnosics performing worse than controls.

#### Discussion

Prosopagnosics showed a significantly reduced face recognition ability compared to controls. This result reflects the impaired holistic face processing and face memory of prosopagnosic participants and replicates findings of many previous studies (e.g., Bate et al., 2013; Duchaine et al., 2007a; Rivolta et al., 2012).

### CCMT

#### Motivation

The CCMT (Dennett et al., 2011) is a test similar in format and structure to the CFMT. We used the CCMT to test for potential general object recognition deficits. We did not expect to find recognition deficits for prosopagnosics in this control task, as only few prosopagnosics might show object recognition deficits which are less severe than their face recognition deficits (Kress & Daum, 2003; Le Grand et al., 2006).

Dennett and colleagues found a significant correlation between the scores of their CCMT and participants’ general interest in cars and knowledge of car makes and models. Therefore, we ran an additional test for car expertise after completing the CCMT, to be able to account for this possible influence and correct the CCMT scores for car expertise.

#### Stimuli and task—CCMT

As a detailed description is given in the original study (Dennett et al., 2011), we give only a short description here. The experimental design is similar to the CFMT, with pictures of whole cars serving as stimuli. The participants were familiarized with six target cars, which they then had to recognize among distractor cars in a three-alternative-forced-choice task. Difficulty was increased stepwise during the test by changing viewpoints and lighting conditions and adding noise. Participants had to decide for each image whether the car had been seen before or not by pressing the relevant keys on the keyboard. The next image appeared as soon as an answer was entered. No feedback was given and no time restrictions were applied. The test can be run in an upright and inverted condition. We only used the upright condition.

#### Stimuli and task—Car expertise

**Figure 2.**
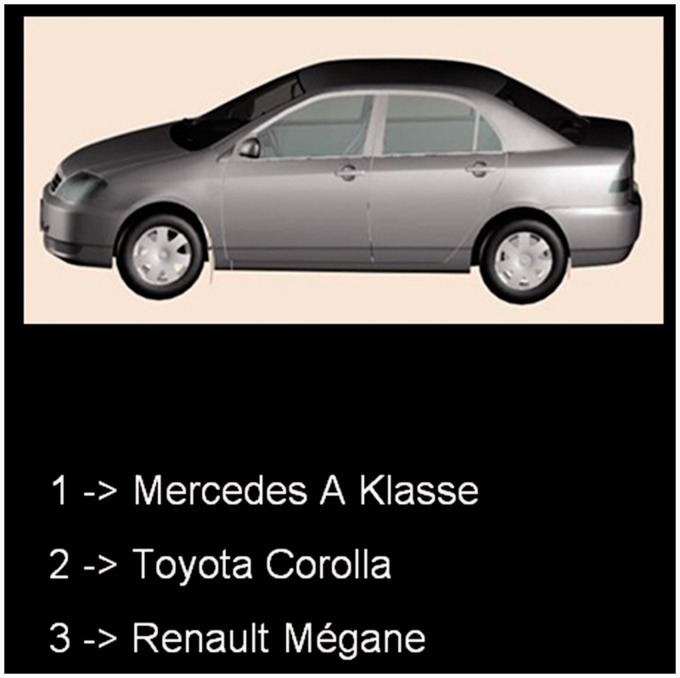
Example trial of the car expertise set. Participants had to pick the correct answer among three written car names.

Sixteen cars from the CCMT (four target and twelve distractor cars) were presented one after the other to the participants along with three answer choices of possible car makes and models (see Figure 2). Participants had to indicate the correct answer by pressing the relevant keys on the keyboard. The next image appeared as soon as a response was entered. No feedback was given and no time restrictions were applied.

The car images in both tests had a visual angle of 5.7° horizontally and 11.4° vertically.

#### Results

**Figure 3.**
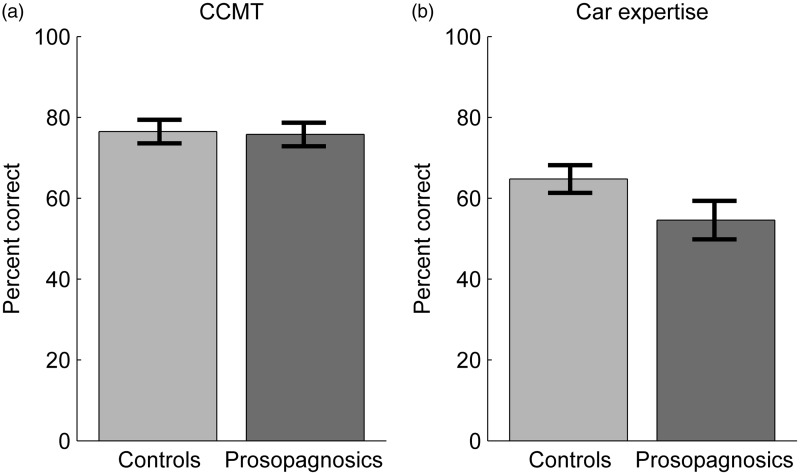
(a) Mean percentage of correctly recognized cars in the CCMT for controls and prosopagnosics. Error bars: *SEM*. (b) Mean percentage of correctly identified cars models for controls and prosopagnosics. Error bars: *SEM*. CCMT = Cambridge Car Memory Test.

The performance measure in both tasks was the percentage of correctly recognized cars per participant. Figure 3 depicts the mean scores per group and task. For the CCMT, the control participants correctly recognized 77.5% (*SD* = 12.9) of the cars, and prosopagnosics scored 75.1% (*SD* = 12.7). For the car expertise test, controls correctly identified 64.6% (*SD* = 16.1) of the car makes, and prosopagnosics scored 52.0% (*SD* = 19.9).

For the CCMT, there was no significant difference in scores between prosopagnosics and controls (one-way-ANOVA, *F*(1, 36) = 0.31, *p* = .58, *η*^2^ = .01). For the car expertise, test the control group exhibited significantly greater expertise in car models than the prosopagnosics (*F*(1, 36) = 4.57, *p* = .04, *η^2^* = .12). Therefore, we compared both groups’ CCMT scores while controlling for the car expertise. For this, we ran a linear regression with car expertise scores as predictor. The residuals of the regression did not differ significantly between groups (one-way-ANOVA, *F*(1, 36) = 0.64, *p* = .43, *η*^2^ = .02), indicating that the CCMT scores do not differ between groups after controlling for car expertise. (Combination of both groups’ regression model was possible, as groups’ regression coefficients were not significantly different from each other (*t*(35) = –0.33, *p* = .75, *d* = –0.11).)

#### Discussion

We found no difference in car recognition performance on the CCMT between controls and prosopagnosics on the groupwise level. This replicates findings of previous studies (McKone et al., 2011; Shah, Gaule, Gaigg, Bird, & Cook, 2015). Even though our control group contained significantly more car experts, we also could not find significant differences in the CCMT scores between controls and prosopagnosics after correcting for car expertise. Furthermore, given the fact that no prosopagnosic scored less than 1.81 *SD* below the mean recognition performance of controls for the CCMT (see Table 3), there was no indication that our prosopagnosic participants had general object recognition deficits, at least in our laboratory conditions.

### Surprise Recognition Test

#### Motivation

Because of their difficulty at recognizing faces, prosopagnosics rely on compensatory strategies to identify people. They report using voice, hairdo, blemishes, or individual forms of face features (Dalrymple et al., 2014; Grüter, Grüter, & Carbon, 2011; Mayer & Rossion, 2009) and use similar strategies in face recognition tasks in laboratory conditions (Duchaine, Parker, & Nakayama, 2003). We developed a test designed to try to bypass these strategies. In the first part of our test, participants were first asked to name facial expressions performed by various actors (implicit learning phase), thus directing their focus to the facial expressions rather than to the identity of the actors. Afterwards, participants had to complete a surprise recognition task of the actors’ faces. Thus, at test we expected to measure prosopagnosics’ face recognition abilities without the interference of their usual strategies, as they did not focus on detecting identification-helping characteristics during implicit learning. This first part was followed by a second, control part with a similar paradigm, but with the difference that participants knew that a face recognition test would follow the presentation of the facial expressions (explicit learning phase). If prosopagnosics did not engage their usual compensatory strategies to remember the faces during the implicit learning phase (first part) but did so during the explicit learning phase (second part), we would expect them to show better performance at test after explicit learning. More importantly, we would expect prosopagnosics to exhibit a stronger recognition improvement between the two test parts than the control group, because then prosopagnosics could actively use their strategies to compensate their impaired holistic processing, while we expected controls to engage holistic processing in both parts.

#### Stimuli

**Figure 4.**
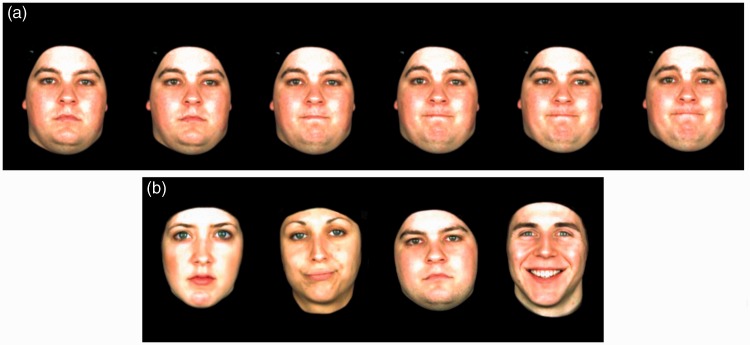
(a) Some consecutive frames of a video of an actor showing the facial expression “I don’t know.” (b) Example stimuli for the test phase: Static images used for testing the participants after training with dynamic videos.

The stimuli were derived from videos from our in-house facial expression database (Kaulard, Cunningham, Bülthoff, & Wallraven, 2012). The database consists of videos of male and female actors performing different emotional and conversational facial expressions (e.g., disgust, considering, being annoyed, etc.) without speaking. Frames extracted from one of the expression videos are shown in Figure 4(a).

A set of 16 videos was used for the implicit learning phase and another set for the explicit learning phase. In each set, four different target actors (two male and two female) were depicted, each showing four different facial expressions. Both the exhibited expressions and the actors’ identities differed in both sets. The videos had a mean length of 2.7 s (*SD* = 1.5). In each test phase, we used 16 static images of the target actors (see Figure 4(b)). These images were taken from different videos not presented to the participants before. As distractors, we used 16 static images taken from 16 new videos with new actors (four images each for two male and two female distractors). All videos and images were frontal views of the faces and had a visual angle of 4.8° horizontally and 6.7° vertically. Different expressions and actors were shown in the first and second part to avoid interference. The assignment of the targets and distractors to the first or second part of the experiment was randomized across participants.

#### Task

In the first part, during the implicit learning phase, participants saw 16 videos: four target actors (two male and two female), each performing four different facial expressions that participants had to name. The order of the videos was pseudorandom such that no actor was seen twice in a row. Participants had to start each video per key press and could watch it only once. After each video, they typed in their interpretation of the facial expression (maximum 80 characters). No feedback was given. After this implicit learning phase, participants performed a surprise old–new recognition task. For this, the participants saw 32 different images: Four images from each of the four target actors and four images from four new distractor actors. Participants had to decide for each image whether the actor had been seen during the learning phase or not by pressing the relevant keys on the keyboard. Stimuli were presented for 2 s or until key press, whichever came first. The next image appeared as soon as an answer was entered. The order of the pictures was pseudorandom, such that no actor was seen twice in a row. No feedback was given. All participants reported that they had not anticipated the surprise recognition task after the expression naming.

The second part was conducted to control for the effect of surprise. The design was similar, with the difference that participants knew that an old–new recognition task would follow the explicit learning phase. Again, the participants watched 16 videos of four different actors. This time they did not need to name the facial expressions but could concentrate on remembering the appearance of the actors. Afterwards they once more had to recognize the actors among the distractors.

#### Results

**Figure 5.**
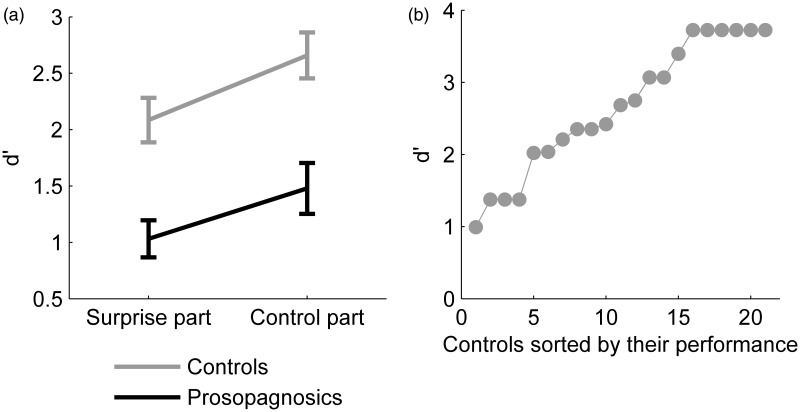
(a) Mean *d*′-scores in the surprise recognition task for controls and prosopagnosics. Error bars: *SEM*. (b) Ceiling effects for the control participants in the second part of the surprise recognition task.

For each participant, we calculated the *d*′-scores as Z(hits)—Z(false alarms). Figure 5(a) depicts the mean scores per group. Controls achieved a mean *d*′-score of 2.09 (*SD* = 0.88) in the first, surprise part and 2.66 (*SD* = 0.91) in the second part. Prosopagnosics achieved a mean *d*′-score of 1.03 (*SD* = 0.64) in the first part and 1.48 (*SD* = 0.87) in the second part. A two-way repeated measures ANOVA of the factors participant group (prosopagnosics, controls) and test part (first, second) was conducted on the *d*′-scores. Recognition performance was significantly higher in the second part compared to the first, surprise part (*F*(1, 35) = 7.1 *p* = .012, *η*^2 ^= .17) and controls performed significantly better than prosopagnosics (*F*(1, 35) = 29.9, *p* < .001, *η*^2 ^= .46). The interaction between parts and participant groups was not significant (*F*(1, 35) = 0.11, *p* = .75, *η*^2^ < .01).

Prosopagnosics and controls performed significantly above chance level (prosopagnosics for both parts *t*(15) > 6.4, *p* < .001, *d* > 1.61; controls for both parts *t*(20) > 10.8, *p* < .001, *d* > 2.48). However, ceiling effects were present for the controls in the second part, as 33% of the controls scored above 95% accuracy (≤one error, *d*′-score ≥ 3.39), 52.4% scored above 90% accuracy (≤three errors, *d*′-score ≥ 2.68)), see Figure 5(b).

#### Discussion

Overall, controls discriminated between old and new faces significantly better than the prosopagnosics in both parts. Importantly, we could not find a difference between groups in their performance improvement in the second part as shown by the absence of an interaction. This finding indicates that, contrary to our prediction, prosopagnosics did not exhibit a stronger recognition improvement between the two test parts compared to controls (e.g., by adapting their strategy). The ceiling performance of the controls reinforces this observation: It may have led to underestimate the improvement between test parts for controls, yet the improvement for prosopagnosics between test parts was still not bigger than for the controls. Because prosopagnosics’ performance was significantly above chance level in the first, surprise part, we conclude that they extracted and stored identity-relevant information even when not paying attention to that information. We suggest two equally possible explanations. First, contrary to our hypothesis, prosopagnosics had engaged their strategies not only during the explicit but also during the implicit learning phase. They were thus able to extract and store featural characteristics even without conscious effort. The second possible explanation is that prosopagnosics’ recognition system does not differ fundamentally from that of the controls in so far that in both groups the mechanisms of holistic processing and extracting identification-relevant information seem to occur automatically in explicit as well as in implicit learning conditions. These automatic mechanisms are exhibited by prosopagnosics, yet are reduced compared to controls. In our next experiment, we investigate whether indeed holistic processing abilities are still present, though in reduced form in prosopagnosics.

### Composite Face Test

#### Motivation

Several studies state that the key to a well-functioning face recognition system lies in holistic face processing. Holistic processing is defined as the integration of all facial information, for example, shape of nose, mouth, and eyes (features) and their spatial distances (configuration). This information is combined into a whole gestalt, making it harder to process the information individually (Maurer et al., 2002). A classical test for holistic processing is the composite face task. When the top half of one face is combined with the bottom half of a different face, both halves are merged into a new, third identity. The combined face halves are processed holistically as a whole, making it difficult to retrieve the identity of the halves individually. This effect disappears when the halves are misaligned. In the composite face task, participants have to indicate if one half (mostly the top half containing the eyes) is the same in two, consecutively shown composite faces. As the lower half interferes with the perception of the upper half, neurotypical participants make more mistakes when the halves are aligned than when they are misaligned. This effect can also be modulated by the choice of the bottom halves: Neurotypical participants make more mistakes when the bottom halves are incongruent to the top halves (i.e., top halves are identical and bottom halves differ and vice versa) than for the congruent case (i.e., either top halves are identical and bottom halves are identical, or top halves differ and bottom halves differ). Our expectations were that in this task evidence of holistic processing would be generally weaker for prosopagnosics than for controls.

We used the “complete design” version of this experiment (Cheung et al., 2008). In the complete design, holistic processing is indexed by an interdependence of congruency and alignment: Performance is better in congruent than in incongruent trials (i.e., congruency effect). Misalignment reduces the congruency effect, as it disrupts holistic processing. We use this version of the experiment because it has been suggested that it may better separate face-specific from non-face-specific effects than the “classic” design (for recent findings supporting this view, see Meinhardt, Meinhardt-Injac, & Persike, 2014, but controversy about this question is ongoing, see e.g., Rossion, 2013). Following McKone and colleagues’ advice (McKone et al., 2013), we tested the composite face effect in upright and inverted conditions. The inverse condition, like misalignment, also disrupts holistic processing. Therefore, inversion in interdependence with congruency also measures holistic processing: The congruency effect is larger for upright than inverted trials.

#### Stimuli

**Figure 6.**
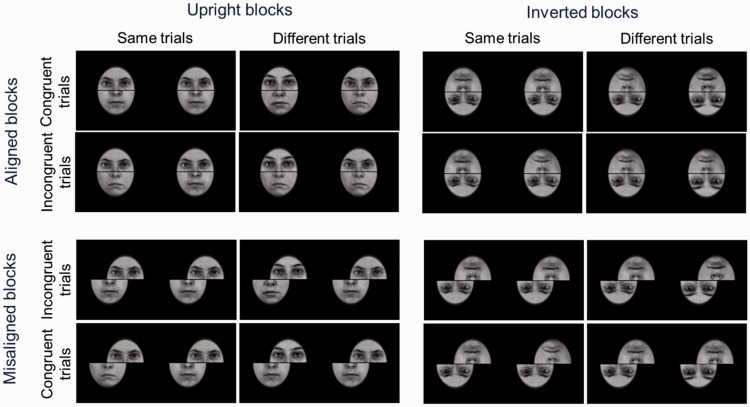
Example stimuli of the composite face task.

The stimuli were created from 12 images of female faces taken from the in-house 3D face database (http://faces.kyb.tuebingen.mpg.de/; Troje & Bülthoff, 1996; Vetter & Blanz, 1999). All images were gray-scale and luminance-equalized, so that the upper and lower half of different faces could be combined without obvious color or luminance differences. To create composites, the faces were cut into top and bottom parts along the center of the image. Bottom and upper face halves were rearranged according to the design of the experiment described below. The composite faces were surrounded with an oval, black mask to cover differences in the outer face shape. Moreover, a horizontal, two pixels thick, black line covered the border between the two halves (see Figure 6). The faces were presented with a visual angle of 2.9° horizontally and 3.8° vertically.

In each trial, two composite faces were presented sequentially for 0.3 s each with an inter-stimulus interval of 0.4 s. The inter-trial interval was 2 s, resulting in an overall trial length of 3 s. When no face was presented, a fixation cross was shown at the center of the image. Participants were instructed to keep their gaze at the position of the fixation cross all the time, even when a face was presented and the cross was not visible.

For the “same” condition, the top half (comprising the eyes) of the first composite face was the same as the top half of the second face within the same trial. In the “different” condition, the two top halves differed. In the congruent condition, the bottom halves were same if the top halves were same or they were different if the top halves were different. In the incongruent condition, the bottom halves were different if the top halves were the same and vice versa. In the aligned condition, top and bottom halves were placed exactly on top of each other. For the misaligned condition, the top half was displaced to the right, while the bottom part was displaced to the left such that the middle of one half was placed adjacent to the edge of the other half. All face images were presented upright for the upright condition or rotated by 180° for the inverted condition.

The combination of upright or inverted condition with aligned or misaligned conditions was tested in four separate blocks. The block order was balanced across participants. Each of the four blocks contained 120 trials: 30 trials of each combination of same and different trials, and congruent and incongruent trials. The order of trials was randomized.

#### Task

In each trial, participants had to indicate whether the two face halves comprising the eyes were the same or not. Participants responded during the inter-trial interval of 2 s by pressing the relevant keys on the keyboard. No feedback was given. After every 20 trials and also between blocks participants were able to take a self-paced break. Before testing, there were 10 training trials for each of the four different blocks. Blocks were trained in the same order as they would appear during the actual testing.

#### Results

**Figure 7.**
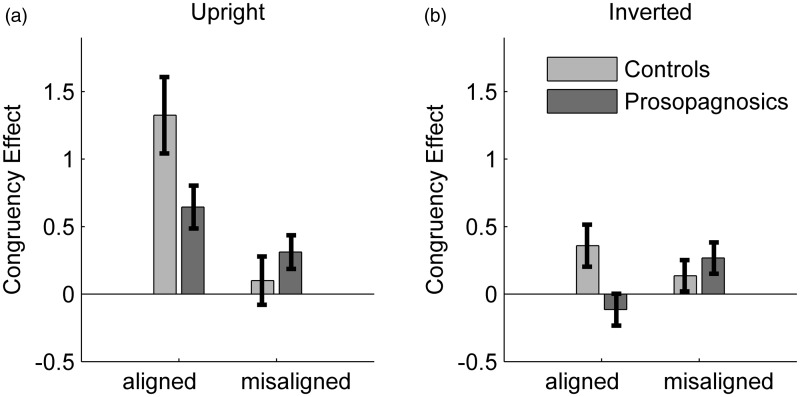
(a) Mean size of the congruency effect in the upright condition for controls and prosopagnosics. Error bars: *SEM*. (b) Mean size of the congruency effect in the inverted condition for controls and prosopagnosics. Error bars: *SEM*.

For each participant we calculated the *d*′-scores as Z(hits = accuracy in same trials)–Z(false alarms = 1−accuracy in different trials). The congruency effect was calculated by subtracting *d*′-scores of incongruent from congruent conditions. Figure 7 depicts the mean congruency effects per group. In the upright condition controls obtained a mean congruency effect of 1.33 (*SD* = 1.27) for aligned and 0.1 (*SD* = 0.8) for misaligned trials, while prosopagnosics obtained a mean congruency effect of 0.65 (*SD* = 0.61) for aligned and 0.31 (*SD* = 0.48) for misaligned trials. In the inverted condition controls obtained a mean congruency effect of 0.36 (*SD* = 0.70) for aligned and 0.14 (*SD* = 0.52) for misaligned trials, while prosopagnosics obtained a mean congruency effect of –0.11 (*SD* = 0.46) for aligned and 0.27 (*SD* = 0.45) for misaligned trials.

As misalignment and inversion are both control conditions for the measurement of holistic processing, we consider these two factors separately. First, we looked at the congruency effect for the upright condition only, using misalignment as control condition. A two-way repeated measures ANOVA on participant group (prosopagnosics, controls) and alignment (aligned, misaligned) was conducted. The congruency effect was larger for the aligned than the misaligned conditions (*F*(1, 35) = 23.54, *p* < .001, *η*^2 ^= .40) and there was no significant difference between participant groups (*F*(1, 35) = 0.93, *p* = .34, *η*^2^ = .03). The interaction between alignment and participant group was significant, indicating that the congruency effect was more affected by misalignment in the control group than for prosopagnosics (*F*(1, 35) = 7.71, *p* = .009, *η*^2 ^= .18). A post hoc analysis for prosopagnosics revealed that their congruency effect was significantly smaller for the misaligned than aligned condition (one-way ANOVA: *F*(1, 15) = 12.90, *p* = .003, *η*^2 ^= .34). This indicates that controls and prosopagnosics exhibit evidence of holistic processing for upright faces.

Second, we looked at the congruency effect for the upright-aligned versus the inverted-aligned conditions only, using inversion as control condition. A two-way repeated measures ANOVA for the aligned condition on orientation (upright, inverted) and participant group (prosopagnosics, controls) was conducted. As expected, the congruency effect was larger for upright than inverted conditions (*F*(1, 35) = 16.0, *p* < .001, *η*^2 ^= .31) and controls showed overall a larger congruency effect than prosopagnosics (*F*(1, 35) = 10.11, *p* = .003, *η*^2 ^= .22). The interaction between orientation and group was non-significant, indicating that the inversion factor did not affect prosopagnosics and controls differently (*F*(1, 35) = 0.23, *p* = .64, *η*^2 ^= .01).

Additionally, we investigated more closely the negative congruency effect observed for prosopagnosics in the inverted-aligned condition (see Figure 7(b)). The congruency effect was significantly smaller for aligned than misaligned trials in the inverted condition for prosopagnosics (*F*(1, 31) = 7.29, *p* = .016, *η*^2 ^= .16). This was not the case for controls, who showed no difference in congruency effects (*F*(1, 41) = 1.27, *p* = .27, *η*^2 ^= .03).

#### Discussion

The congruency effect in interdependence with (a) alignment or (b) orientation serves as a measure of holistic processing. For the upright condition, using (a) misalignment as control condition, we found that controls showed a larger difference in congruency effect for aligned versus misaligned trials compared to prosopagnosics. These results suggest that holistic processing is impaired or utilized to a smaller extent by prosopagnosics in this task. This replicates the results of previous reports of decreased holistic processing for prosopagnosics compared to controls (Avidan et al., 2011; Palermo et al., 2011).^2^ Importantly, the difference in congruency effect for aligned versus misaligned trials is significant for prosopagnosics, which indicates that their holistic processing ability is still present yet impaired.

When we used (b) the inversion effect (upright-aligned versus inverted-aligned conditions only) to assess holistic processing, no significant difference between groups appeared (non-significant interaction). Thus, we did not find differences in holistic processing between groups, which is contrary to the expectations given by our design. Furthermore, our results also imply that prosopagnosics show more holistic processing for misaligned face halves than aligned halves when seen inverted. Similar “inversion superiority effects” for prosopagnosics have been described before (Behrmann, Avidan, Marotta, & Kimchi, 2005; de Gelder, Bachoud-Lévi, & Degos, 1998; Farah, Wilson, Maxwell Drain, & Tanaka, 1995). However, what exactly happens when prosopagnosics process inverted faces is currently not well understood. For this reason, the results in the inverted condition should not be seen as a true indicator of holistic processing for prosopagnosics. We would argue that the advice to run the composite face effect in upright and inverted conditions (McKone et al., 2013) is not suitable for testing prosopagnosics. Nevertheless, the fact that the controls showed the expected pattern in the inverted condition (small congruency effects with no difference between alignment conditions) supports the general validity of this method.

In sum, if we concentrate on the upright condition that can be clearly interpreted, prosopagnosics, compared to controls, show a smaller difference between the congruency effects obtained in the aligned and misaligned condition. This indicates that holistic processing (as indexed by the interdependence of congruency and alignment) is present but impaired for prosopagnosics.

The composite task is generally regarded to be the best method to assess holistic face processing. There are many other methods (see e.g., Piepers & Robbins, 2012) which are used to investigate holistic processing. Whether they measure the exact same mechanisms as those involved in the composite task and how those different mechanisms might relate to one another are still open questions. To get a closer and more detailed look at the impairment of holistic processing in prosopagnosia, we tested what type of facial information retrieval is impaired in the study described next.

### Featural and Configural Sensitivity Test

#### Motivation

Our test using the composite face task revealed that holistic processing is impaired in prosopagnosics. It is debated whether holistic processing relies exclusively on configural processing or whether featural appearance (part-based contribution) might be involved as well and in what way (Goffaux & Rossion, 2006; McKone, 2004; McKone & Yovel, 2009; among others). Investigating prosopagnosics’ sensitivity to configural and featural facial information might shed some light on this issue. To that end, we generated a stimulus set of natural looking faces with parametric differences in features and configuration for a fine-grained investigation of the sensitivity of prosopagnosics and controls to featural and configural facial information.

Stimulus creation and task have been described in details elsewhere (Esins, Schultz, Wallraven, & Bülthoff, 2014). Therefore, we will give only a short description here.

#### Stimuli

**Figure 8.**
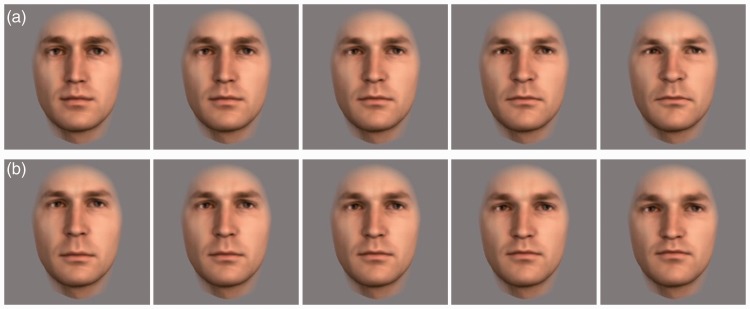
Faces of one set, (a) differing in features while their configuration stays the same and (b) differing in configuration while their features stay the same. Skin texture and outer face shape were kept constant within each set. The middle faces of both rows are the same.

We manipulated male faces from our in-house 3D face database to create eight face sets. Different faces were used for each set. In each created set, the faces differed in features (eyes, nose, and mouth) or their configuration, but they shared the same skin texture and outer shape (see Figure 8). Skin texture and outer shape of each set differed from the others. Changes in features and in configuration were implemented parametrically, resulting in five similarity levels from 100% (identical faces) to 0% (maximal difference within each set) between the faces. The central faces of both dimensions (features and configuration) are identical for each set. In a previous study (Esins, Bülthoff, & Schultz, 2011), the natural appearance of these faces has been controlled.

The stimuli had a visual angle of 5.7° horizontally and 8.6° vertically. To prevent pixel matching, the faces were presented at different random positions on the screen within a viewing angle of 7.6° horizontally and 10.5° vertically.

#### Task

Participants rated the perceived pairwise similarity of the faces within each set on a Likert scale from 1 (very little similarity) to 7 (high similarity/identical). They were advised to use the whole range of ratings during the experiment. In each trial, the first face was displayed for 2 s, followed by a pixelated face mask for 0.8 s, and then the second face for another 2 s. Afterwards, the Likert scale was displayed and participants marked their rating by moving a slider on the scale via the arrow keys and confirmed their choice by pressing the relevant key on the keyboard. The start position of the slider was randomized. The next trial started as soon as the rating was confirmed. There were no time restrictions, but participants were told to answer without too long considerations. After every 20 trials, participants could have a self-paced break.

The faces of each set were compared with each other and with themselves. We were only interested in trials comparing faces manipulated along the same dimension (see Figure 8(a) for features and (b) for configuration). Filler-trials in which faces differed in both features and configuration were displayed during the test to avoid participants realizing the nature of the stimuli. These filler-trials were omitted from the analysis. For each participant, the order of trials was randomized within and across sets.

#### Results

For each participant, we calculated the mean ratings for each of the five similarity levels across all sets, but separately for each change type (featural, configural). Similarity ratings were close to seven (high similarity) for identical faces and dropped with decreasing similarity. For each participant, we fitted a linear regression to the mean similarity ratings, again separately for featural and for configural ratings. The similarity levels served as predictors. The steepness of the slopes was then used as measure of sensitivity: The steeper the slopes, the stronger the participant perceived the configural or featural changes.

**Figure 9.**
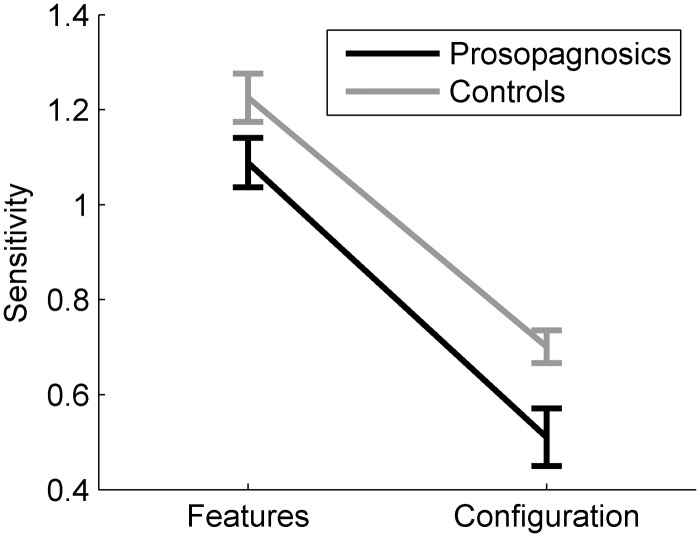
Mean sensitivity to features and configuration for controls and prosopagnosics. Error bars: *SEM*.

Figure 9 depicts the mean sensitivity scores per group. Controls obtained a mean sensitivity score of 1.23 (*SD* = 0.23) for featural and 0.70 (*SD* = 0.15) for configural changes. Prosopagnosics obtained a mean sensitivity score of 1.09 (*SD* = 0.20) for featural and 0.51 (*SD* = 0.23) for configural changes. We analyzed the sensitivity scores with a two-way repeated-measures ANOVA with the factors change type (features, configuration) and participant group (prosopagnosics, controls). Participants exhibited a higher sensitivity towards featural than configural changes (*F*(1, 35) = 172.76, *p* < .001, *η*^2 ^= .83), and prosopagnosics showed an overall reduced sensitivity compared to controls (*F*(1, 35) = 9.34, *p* = .004, *η*^2 ^= .21). The interaction between change type and participant group was non-significant (*F*(1, 35) = 0.41, *p* = .53, *η*^2 ^= .01).

#### Discussion

The use of parametric stimuli in our tests allowed a more fine-grained quantification of potential configural and especially of featural processing impairment in prosopagnosics than most other studies. We morphed facial features (eyes, nose, and mouth) between two faces in several steps in each set in terms of their color and shape, thus creating faces differing gradually for these features. In contrast, most other studies only exchanged features between faces. Furthermore, we took care to use faces without make-up and all our stimuli had been controlled for their naturalness (for details, see Esins, Bülthoff, & Schultz, 2011; Esins, Schultz, et al., 2014).

With these stimuli, our results show that the sensitivity to configural and featural information is significantly impaired for prosopagnosics compared to controls. It is nevertheless worth noting that we carried out a more detailed analysis of our findings in the framework of a previous study (Esins, Schultz, et al., 2014) that was based partly on the data presented here. That analysis had shown that configural sensitivity was significantly impaired, while featural sensitivity was only marginally significantly impaired for prosopagnosics in comparison to controls. This finding might indicate that featural information in congenital prosopagnosics is less clearly impaired than configural information.

For comparison, Le Grand and colleagues found prosopagnosics to have a significantly lower accuracy than controls for faces modified in configuration, but not for faces modified in features (Le Grand et al., 2006). This result was confirmed by Yovel and Duchaine (2006) who used the same stimuli to test prosopagnosic participants. However, these authors criticized these stimuli for the presence of make-up increasing the saliency of the eyes and lips besides the manipulation of the shape and the distance between features. This critique was addressed in a study that used faces without make-up (Mondloch, Robbins, & Maurer, 2010), which confirmed the higher sensitivity to features than to configuration. When Yovel and Duchaine (2006) tested their participants with other faces wearing no make-up, they found that prosopagnosics showed a reduced sensitivity to both types of information (Yovel & Duchaine, 2006). Note, though, that their face stimuli without make-up were criticized for having configural modifications beyond natural limits (as discussed in Maurer et al., 2007). It was also shown that prosopagnosics obtained significantly lower recognition scores than controls for both featural and configural information in another study using blurred (disrupted featural information with intact configural information) and scrambled (disrupted configural information with intact featural information) face stimuli (Lobmaier et al., 2010).

The results of the composite face test and the featural and configural sensitivity test indicate that not only holistic processing but also the retrieval of configural information is impaired in prosopagnosics. Further, the retrieval of featural information might be impaired to a lesser degree than configural information as indicated by our previous study based on the same stimuli. In sum, the results of the composite face test and the featural and configural sensitivity test in this study support the view that deficits in holistic processing in congenital prosopagnosia are due to deficits not only in configural but also at least in part, in featural processing.

### Gender Recognition Test

#### Motivation

Most prosopagnosics self-report normal recognition of the gender of faces (Grüter, Grüter, & Carbon, 2008) which is also reflected by the results of behavioral studies (Chatterjee & Nakayama, 2012; DeGutis, Chatterjee, Mercado, & Nakayama, 2012; Le Grand et al., 2006). Nevertheless, there are some single-case studies which report prosopagnosics’ gender recognition to be impaired (Ariel & Sadeh, 1996; De Haan & Campbell, 1991; Duchaine, Yovel, Butterworth, & Nakayama, 2006; Jones & Tranel, 2001). In view of those conflicting reports, we aimed at clarifying this issue.

#### Stimuli

**Figure 10.**
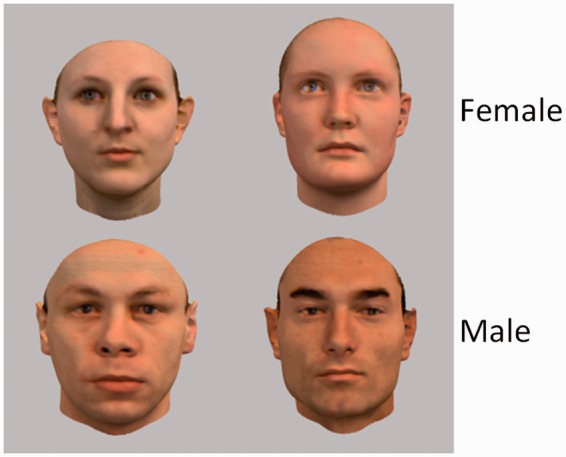
Example of female and male faces used as stimuli for the gender recognition task.

We used 80 faces (40 male) from our in-house 3D face database. As visible in Figure 10, the faces contained no extra-facial cues (e.g., hair, beard, or makeup) about their gender. The stimuli had a visual angle of 3.8° horizontally and 5.7° vertically.

#### Task

Participants had to judge the gender of each face. The faces were shown one at a time and stayed on screen until a response was given by pressing the relevant keys on the keyboard. The next image appeared as soon as a response was entered. The order of trials was randomized. No feedback was given. Participants were instructed to answer as correctly and as quickly as possible.

#### Results

**Figure 11.**
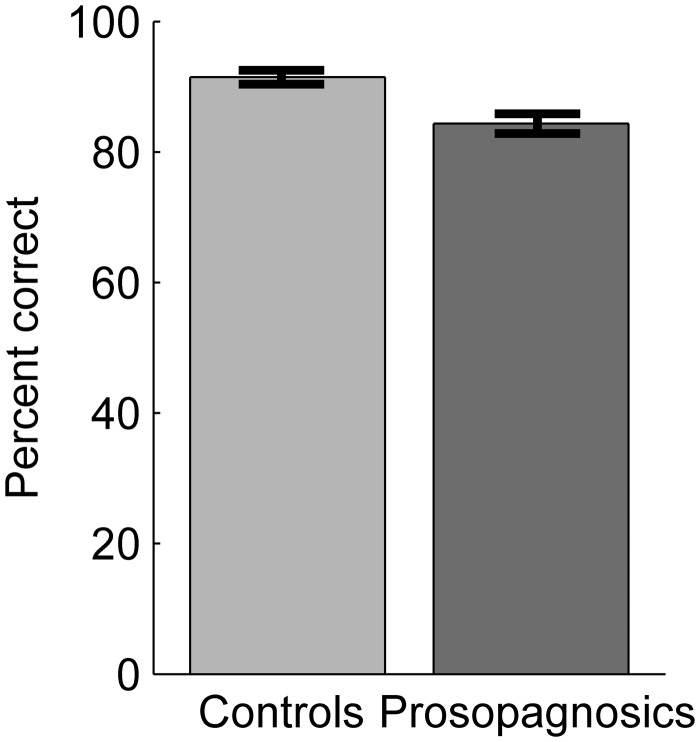
Mean percent correctly classified faces in the gender recognition task for controls and prosopagnosics. Error bars: *SEM*.

For each participant, percent correct accuracy was calculated. Figure 11 depicts the mean scores per group. Controls achieved a very high mean accuracy of 91.5% (*SD* = 4.8), while prosopagnosics scored very well too at 84.4% (*SD* = 5.9). Nevertheless, prosopagnosics performed significantly worse than controls as revealed by a one-way ANOVA (*F*(1, 36) = 16.62, *p* < .001, *η*^2 ^= .32).

#### Discussion

Prosopagnosics exhibited a significantly lower gender recognition ability compared to controls. This differs from the self-reports of prosopagnosics (Grüter et al., 2008) and also from behavioral tests in several studies (Chatterjee & Nakayama, 2012; DeGutis et al., 2012; Le Grand et al., 2006). However, there are some single case studies of prosopagnosics which report impairments of gender recognition (Ariel & Sadeh, 1996; De Haan & Campbell, 1991; Duchaine et al., 2006; Jones & Tranel, 2001). But to the best of our knowledge, our study is the first to report an impairment in gender recognition on a groupwise level for prosopagnosics. In our test, we observed high performance for the control group and comparatively high performance for the prosopagnosics. We argue that first, prosopagnosics suffer from only a slight impairment of gender recognition and second, that this impairment may be easily compensable in daily life by using cues like body shape, hairdo, makeup, voice, etc. Our conclusion that an impairment in gender recognition is only slight and easily compensable is supported by the fact that controls and prosopagnosics achieved ceiling performance in gender recognition tests in several further studies (Dobel, Bölte, Aicher, & Schweinberger, 2007; Gruber, Dobel, Jungho, & Junghöfer, 2011; Lobmaier et al., 2010). In our study, we used well-controlled stimuli derived from real faces. It is possible that this type of stimuli and our large sample size helped to reveal the gender recognition deficit in prosopagnosics. Along this line, another study which also used faces of the same 3D face database showed impaired same-or-different recognition performance for faces differing in gender for their prosopagnosic participants (Behrmann et al., 2005).

### Facial Motion Advantage Test

#### Motivation

Most studies testing holistic face recognition abilities of prosopagnosics use only static face stimuli. Furthermore, those face images often are identical for training and testing. Such tasks do not reflect the everyday challenges encountered by prosopagnosics, as people move, speak, and might alter their look on a day-to-day basis. On the one hand, the different looks of people might complicate recognition for prosopagnosics maybe even more than for controls who do not rely on these non-facial attributes for recognition. On the other hand, the additional dynamic information might give additional cues for prosopagnosics, thus facilitating recognition (*motion advantage*). Again, this advantage may potentially be even stronger for prosopagnosics than for controls who do not need to rely on this additional information for recognition. Therefore, we wanted to investigate the influence of appearance (e.g., hairstyle, makeup) and motion on face recognition for prosopagnosics compared to controls, by using dynamic stimuli in which the same people changed their look between learning and test. We also included static stimuli and faces that did not change their appearance (identical stimuli) as control conditions. We expected that, for the identical stimuli, the prosopagnosics would retain their usual compensatory strategies, while for the changed stimuli they would make more use of the dynamic information.

#### Stimuli

**Figure 12.**
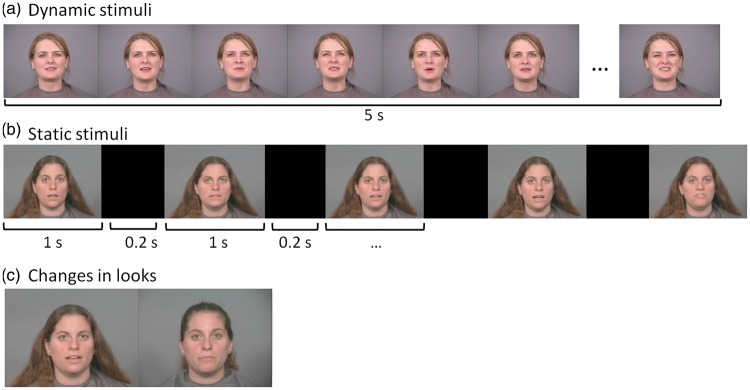
Example of stimuli. (a) Dynamic stimuli: recordings of persons speaking. (b) Static stimuli: five random frames extracted from the original recordings are shown one after the other. Each frame is shown for 1 s with a black frame for 0.2 s between frames. (c) Example of difference between recordings for the changed condition.

The stimuli used in this test have been created and kindly provided by O’Toole and colleagues (O’Toole et al., 2005) and only a short description is given here. Recordings of 72 actresses speaking into the camera, expressing natural rigid and non-rigid movements served as dynamic stimuli (Figure 12(a)). The static stimuli displayed five random frames from the original recordings, shown for 1 s each and separated by a black screen for 0.2 s (Figure 12(b)). Actresses were shown only in one of both conditions (static or dynamic) during the task. There were two recordings of each actress. In the second recordings, the actresses had a different hairdo, makeup, or accessories (see Figure 12(c)). These different recordings with a changed look were also prepared as dynamic and static stimuli, respectively.

All stimuli presented the faces for 5 s and were mute. Each actress was placed in front of a gray background and her clothing was covered. The stimuli had a visual angle of 16.6° horizontally and 12.4° vertically.

#### Task

The experiment consisted of two blocks. In one block, dynamic stimuli were used for learning and testing, and static stimuli were used in the other block. The block order was counterbalanced between participants. In each block, participants first learned 18 target identities and then performed an old–new recognition test on these 18 target identities intermixed with the same number of distractor identities. Half of the targets were identical during learning and at test (identical stimuli) while for the other half of the targets the second recording was presented at test (changed stimuli). Participants were informed that the look might change between learning and testing. The order of trials was randomized during learning and testing. Which target actresses were tested in the identical or changed condition was counterbalanced across participants. During learning and testing participants could see each stimulus only once. During testing, the stimuli were presented as long as described above or until key press, whichever came first. The next stimulus started as soon as an answer was entered by pressing the relevant keys on the keyboard. No feedback was given. Between blocks participants were able to take a self-paced break.

#### Results

**Figure 13.**
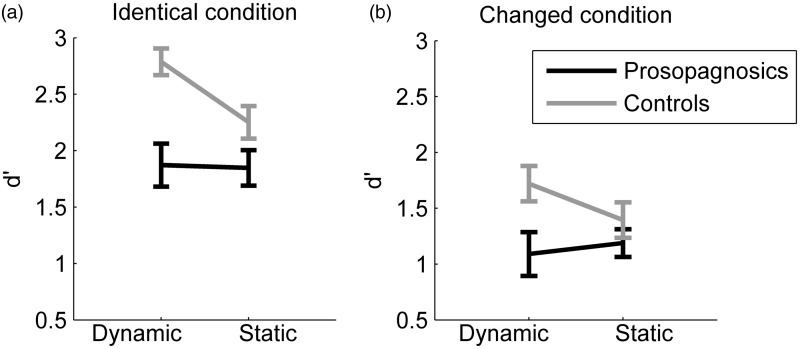
(a) Mean *d*′-scores for the identical condition for controls and prosopagnosics. Error bars: *SEM*. (b) Mean *d*′-scores for the changed condition for controls and prosopagnosics. Error bars: *SEM*.

We calculated *d*′-scores for each participant. Figure 13 depicts the mean scores per group in the identical and the changed condition. For the identical condition, controls achieved a mean *d*′-score of 2.79 (*SD* = 0.53) for dynamic stimuli and 2.25 (*SD* = 0.65) for static stimuli. Prosopagnosics achieved a mean *d*′-score of 1.87 (*SD* = 0.74) for dynamic stimuli and 1.85 (*SD* = 0.61) for static stimuli. For the changed condition, controls achieved a mean *d*′-score of 1.72 (*SD* = 0.71) for dynamic stimuli and 1.40 (*SD* = 0.71) for static stimuli. Prosopagnosics achieved a mean *d*′-score of 1.09 (*SD* = 0.76) for dynamic stimuli and 1.19 (*SD* = 0.48) for static stimuli.

We ran a two-way repeated measures ANOVA for the identical and changed condition versus participant group (prosopagnosics, controls). We found a significantly better performance in the identical condition (*F*(1, 35) = 117.32, *p* < .001, *η^2^*^ ^= .77) and a significantly better performance for controls (*F*(1, 35) = 15.46, *p* < .001, *η*^2 ^= .31). The interaction was non-significant (*F*(1, 35) = 2.42, *p* = .13, *η*^2^ = .07), indicating that there was no difference between controls and prosopagnosics in how the changed appearance of the faces influenced their performance.

We also conducted two-way repeated measures ANOVAs on stimulus type (dynamic, static) and participant group (prosopagnosic, control) for the identical and for the changed condition separately. For the identical condition, we found a statistically better performance for dynamic than static stimuli (*F*(1, 35) = 4.99, *p* = .032, *η*^2 ^= .13) and a statistically better performance for controls than prosopagnosics (*F*(1, 35) = 15.62, *p* < .001, *η*^2 ^= .31). Interaction between stimulus type and participant group was significant (*F*(1, 35) = 4.14, *p* = .0496, *η*^2 ^= .11). Post hoc analysis of simple effects of stimulus type (dynamic, static) revealed that controls performed better for dynamic than static stimuli in the identical condition (one-way ANOVA, *F*(1, 41) = 8.65, *p* = .005, *η*^2 ^= .18), while there was no difference between dynamic and static stimuli for prosopagnosics (*F*s = 0.02, *p* = .92, *η*^2^ < .01).

For the changed condition, there was no performance difference for static versus dynamic stimuli (two-way ANOVA, *F*(1,35) = 0.44, *p* = .51, *η*^2 ^= .01). We found a better performance for controls than prosopagnosics (*F*(1,35) = 8.03, *p* = .008, *η*^2 ^= .19). The interaction between stimulus type and participant group was non-significant (*F*(1, 35) = 1.54, *p* = .22, *η*^2 ^= .04).

#### Discussion

The first finding of this test is that controls and prosopagnosics showed a similar drop in recognition performance when the appearance of a person changes compared to the identical condition. Therefore, with this design, we did not find evidence that prosopagnosics are more affected than controls when unfamiliar faces change appearance between learning and testing.

Second, we found that controls, but not prosopagnosics, showed a facial motion advantage when tested with identical stimuli. These results are in line with a study which also tested prosopagnosics with dynamic and static face stimuli in an old–new recognition task with faces presented either upright or inverted (Longmore & Tree, 2013). In that study, prosopagnosics showed no significant difference in performance for dynamic and static stimuli in the upright condition, while controls performed better for the dynamic stimuli. Longmore and colleagues’ interpretation was that the task was too difficult for the prosopagnosics, making it impossible to detect a facial motion advantage for this group because of a floor effect (mean accuracy rates of the prosopagnosics were about 60% for both static and dynamic upright stimuli, with the chance level being 50%). Similarly, we find no motion advantage for prosopagnosics in the identical condition. However, our task did not seem to be too difficult: Prosopagnosics showed mean *d*′-scores between 1.0 and 2.0, with *d*’ = 0 corresponding to chance level. Therefore, we argue that our results provide a valid measure of the absence of a motion advantage for prosopagnosics.

In the changed condition, when appearance changes between learning and test, both groups showed no difference in recognition performance between static and dynamic stimuli. This is contrary to our expectations that prosopagnosics would rely more on dynamic information than controls in this condition. It is worth noting that, in an earlier report of that test, we had found a significant interaction between participant group and motion information, with a significant motion advantage for controls in both conditions (Esins, Bülthoff, & Schultz, 2014). At that time, we had analyzed recognition performance of the same 16 prosopagnosic participants and 16 of the 21 controls reported here, matched to the prosopagnosics in age and gender as closely as possible. Therefore, we suggest, that a larger sample size is needed to verify the robustness of this finding.

Further support for the lack of a motion advantage for prosopagnosics reported here is given by a study reporting impaired biological motion perception for face, but not whole-body stimuli for congenital prosopagnosic participants (Lange et al., 2009).

Taken together, these previous studies and our results hint at a lack of a motion advantage for prosopagnosics. This could be explained by a neurophysiological dysfunction in prosopagnosia that affects not only the ventral temporal face-processing regions but also the lateral temporal facial motion-processing regions, in particular the superior temporal sulcus (Hoffman & Haxby, 2000), a core region of the face processing network (Ishai, Schmidt, & Boesiger, 2005). The right posterior superior temporal sulcus (STS) was found to have a significantly reduced connectivity with the other core regions of the face processing network in prosopagnosics (Avidan et al., 2013). This finding is in accordance with the fact that we find no motion advantage for prosopagnosics tested in our study.

## Reliabilities

As our study included newly developed tests, we assessed their reliability by calculating the reliability scores for each participant group separately. The reliability is an indicator of the test’s internal consistency quality: In a test with high reliability, all trials involving the same face recognition mechanisms give similar results. We calculated the internal consistency reliability with Cronbach’s alpha. For consistency reasons, this was done for all tests, also for the established tests CFMT and CCMT, especially as this has never been calculated for prosopagnosic populations. However, for the surprise recognition test and the facial motion advantage test, Cronbach’s alpha could not be calculated, because participants saw different stimuli, depending on assignment. For this reason, we also calculated the reliability coefficient with a bootstrapped split-half method with Spearman–Brown correction. This method is mathematically related to Cronbach’s alpha and can be applied to all our tests. Both coefficients gave similar results, validating the calculation (see Table 6). Additionally, we calculated the statistical difference between controls and prosopagnosics for either reliability coefficient. For Cronbach’s alpha, this was calculated based on the Fisher–Bonett approach (Bonett, 2003). For the split-half reliability coefficients, it was calculated as statistical difference between correlation coefficients (Fisher, 1921). The *p* values are given in Table 6 as well. Many of our tests comprised of several parts testing different aspects of face recognition (cars test (3.2), surprise recognition test (3.3), composite face test (3.4), featural and configural sensitivity test (3.5), facial motion advantage test (3.7)). Therefore, we calculated Cronbach’s alpha and split-half estimate for the different test parts separately.

**Table 6.** Reliability Coefficients, Statistical Significance of Difference and Ratio Between Groups’ Reliability Coefficients for Each Test.

Tests	Parts	Prosopagnosics		Controls		*p*		Ratio	
		Cronbach’s alpha	Adjusted split-half	Cronbach’s alpha	Adjusted split-half	Cronbach’s alpha	Unadjusted split-half	Cronbach’s alpha	Adjusted split-half
3.1. CFMT		0.26	0.31	0.82	0.83	0.01*	0.05	3.15	2.68
3.2. Cars	CCMT	0.87	0.88	0.88	0.89	0.83	0.89	1.01	1.01
	Car-expertise	0.69	0.72	0.56	0.58	0.49	0.58	0.81	0.81
3.3. Surprise recognition	Surprise	–	0.30	–	0.66	–	0.32	–	2.20
	Control	–	0.65	–	0.76	–	0.62	–	1.17
3.4. Composite face task	Up-algn	0.85	0.86	0.94	0.95	0.04*	0.16	1.11	1.10
	Up-misalgn	0.86	0.87	0.92	0.93	0.27	0.42	1.07	1.07
	Inv-algn	0.86	0.87	0.92	0.92	0.28	0.43	1.07	1.06
	Inv-misalgn	0.83	0.84	0.91	0.92	0.24	0.40	1.10	1.10
3.5. Featural and configural sensitivity task	Features	0.97	0.97	0.94	0.94	0.18	0.35	0.97	0.97
	Configuration	0.97	0.97	0.96	0.96	0.65	0.74	0.99	0.99
3.6. Gender recognition		0.67	0.70	0.69	0.72	0.88	0.91	1.03	1.03
3.7. Facial motion advantage	Dynamic	–	0.52	–	0.44	–	0.83	–	0.85
	Static	–	0.17	–	0.50	–	0.48	–	2.94

CFMT = Cambridge Face Memory Test; CCMT = Cambridge Car Memory Test; up = upright; inv = inverted; algn = aligned; mislagn = misaligned. Reliability coefficients were calculated with bootstrapped Spearman–Brown adjusted split-half method and, where possible, with Cronbach’s alpha. Statistical significance of difference between groups’ reliability coefficients was calculated as difference between correlations for unadjusted split-half reliability coefficients and with the Bonett formula for Cronbach’s alpha (Bonett, 2003). The ratio between prosopagnosics and controls reliability coefficients was calculated by dividing controls reliability coefficients by prosopagnosics reliability coefficients. ^*^ Statistically significant p values

For Cronbach’s alpha, coefficients of more than .7 indicate acceptable to excellent reliability (Lance, Butts, & Michels, 2006; Nunnally, 1978). For controls, reliability coefficients close to .7 and higher (and mostly larger than .9) were reached in most tests parts (see Table 6). For prosopagnosics, most reliability coefficients were similar to those obtained by the controls and deviated by less than 20% (i.e., the ratio of reliability coefficients between groups was between 0.8 and 1.2, see Table 6). However, in four tests or test parts, prosopagnosics’ reliability coefficients conspicuously deviated from controls’ coefficients (CFMT (3.1), the surprise condition of the surprise recognition test (3.3), the upright-aligned condition of the composite face test (3.4), and the static condition of the facial motion advantage test (3.7)). For the surprise recognition test, the facial motion advantage test and the CFMT, controls exhibited more than two to three times higher reliability coefficients than prosopagnosics (i.e., the ratio of reliability coefficients was larger than 2.2. See Table 6). The difference of reliability coefficients between groups reached significance for the CFMT and composite face test, but not for the surprise recognition test and the facial motion advantage test.

A literature search for experimental reliability coefficients for the CFMT found only studies reporting Cronbach’s alpha for control participants: Cronbach’s alpha = .83 (Herzmann et al., 2008), Cronbach’s alpha = .90 (Wilmer et al., 2010), and Cronbach’s alpha = .89 (Bowles et al., 2009). We were not able to find a study reporting reliability for the CFMT for purely prosopagnosic participant groups. Therefore, we report here for the first time this interesting result.

Importantly, all tests reported above for which prosopagnosics showed a conspicuous deviation of their reliability coefficients compared to controls, test for holistic recognition of static faces, that is, all tests in which participants had to recognize the identity of whole static faces. The other tests do not investigate holistic face recognition but rather face classification, featural and configural processing, face parts comparison, object recognition, or deal with moving faces. The fact that there is no reduced reliability for recognition of dynamic faces in the test for the facial motion advantage could have several causes. One possible explanation is that other mechanisms than holistic processing is activated when recognizing dynamic faces, which allows the performance of prosopagnosics to be more consistent. This hypothesis is supported by a study finding that non-rigid face motion promotes part-based processing rather than holistic processing in laboratory conditions (Xiao, Quinn, Ge, & Lee, 2013).

These reliability results lead us to the following hypothesis. The calculated test reliabilities are equivalent to the consistency of response behavior of the participants. It is known that prosopagnosics use compensatory, part-based strategies to bypass their limited face recognition abilities in everyday life, but also in test situations (Dalrymple et al., 2014; Duchaine et al., 2003; Grüter et al., 2011; Mayer & Rossion, 2009). The low reliability could be caused by this use of various strategies. Prosopagnosics might switch between strategies, combine several different strategies, or respond at random if they find that none of their strategies works, thus causing their inconsistent response behavior as measured by the reliability coefficients. This is in line with a study by McKone et al. (2011), testing control participants with the CFMT-Australian in upright and inverted version and finding a reduced reliability for the later condition. These authors also concluded that holistic processing works consistently for upright faces, while for inverted faces a more variable approach of part-based-processing is adopted. As soon as holistic processing is discarded in favor of part-based strategies, the reliability decreases.

However, we want to provide another possible explanation, namely that some internal processes for holistic face recognition do not work consistently for prosopagnosics. Our test results do not allow identifying the exact cause for this reduced reliability. Therefore, further testing is necessary, also to verify the robustness of this finding.

If indeed strategy usage, random answering, or inconsistent internal processes cause the reduced test reliability for prosopagnosics, this raises doubt whether the same perceptual processes and mechanisms are measured for controls and prosopagnosics and also within the prosopagnosics themselves. Because significant performance differences between controls and prosopagnosics were observed in at least one part of all face perception tests, we argue that these tests are suitable for a coarse comparison of face processing abilities between groups, even though for some tests there are apparently qualitative differences in reliability. However, for a more detailed analysis of performance levels, for example, at an individual level, the tests might be too unreliable. In addition, the low reliabilities affect correlation analyses between tests. The correlation between test performances is restricted by the tests’ reliabilities: The square root of the product of reliabilities of two tests gives an upper boundary to their correlation (Nunnally, 1970). Correlation analyses are often used to relate different face perception mechanisms, for example, if face identification performance is linked to holistic processing (Degutis et al., 2013; Konar, Bennett, & Sekuler, 2010; Richler et al., 2011; Zhao, Hayward, & Bülthoff, 2014). It is also used to examine if similar impairments exist in different cases of prosopagnosia (Duchaine, Germine, & Nakayama, 2007; Duchaine, Yovel, & Nakayama, 2007; Kennerknecht et al., 2007). Our finding therefore is very important for the search of systematic patterns of impairment and possible common subgroups among prosopagnosics. As the low reliability for prosopagnosics adds noise to test results, this might complicate the identification of response patterns and subgroups in prosopagnosia, which is an actual focus of prosopagnosia research.

## General Discussion

In the present study, we compared prosopagnosics to controls by assessing their face and object recognition abilities in a variety of tests. The face tests investigated holistic processing, sensitivity to featural and configural information, gender recognition, benefit of motion information, and the unconscious, automatic extraction of identity information, while two additional tests measured participants’ recognition performance for objects. Significant differences in performance between prosopagnosics and controls were observed in all face tests, while both groups did not differ in the object tests. Besides acquiring more detailed descriptions of prosopagnosics’ impairments in face recognition (as discussed in each test section), our study also brings to light some fundamental difference in the quality of the obtained data. It reveals that classical tests engaging holistic processing might not be adequate for prosopagnosic participants although they are well adapted for fine-grained investigations of face recognition in neurotypical populations.

Prosopagnosics in this study displayed reduced face recognition (CFMT) and normal object recognition (CCMT). More interestingly, we found that prosopagnosics showed automatic retrieval of identity information (surprise recognition test), indicating that their unconscious mechanisms for extracting identification-relevant information are still active whenever they view faces. Furthermore, we could replicate the finding that prosopagnosics show limited holistic processing (composite face test), supporting the assumption that holistic processing is decisive for efficient identification (Farah, Wilson, Drain, & Tanaka, 1998). We further refined this result by testing the same groups of participants with the featural and configural sensitivity test, which revealed that especially configural processing, and maybe featural processing to a lesser extent, are impaired in prosopagnosics. These findings reinforce the hypothesis of involvement of featural and configural processing with holistic processing and face identification. Our findings fit very well with recent neuroimaging findings showing abnormal brain activation patterns in both core (fusiform face area (FFA), occipital face area (OFA)) and extended (anterior temporal) face processing regions (Rivolta et al., 2014) as well as disrupted connectivity between those regions (Avidan et al., 2014). In particular, a very recent study revealed impaired configural face processing in the right FFA in prosopagnosics (Zhang, Liu, & Xu, 2015), demonstrating that functionality within a central face processing area is affected in a manner directly visible behaviorally. In addition to our evidence for reduced holistic processing in prosopagnosics, we also showed that they are impaired in gender recognition (gender recognition test), confirming previous findings that not only face identification but also other basic face discrimination tasks like gender discrimination rely on holistic processing (Zhao & Hayward, 2010). To the best of our knowledge, we are the first to show an impairment in gender recognition on a groupwise level. Finally, prosopagnosics did not benefit from facial motion when asked to recognize faces, while controls did (facial motion advantage test). Again, this finding corresponds nicely to the neuroimaging studies mentioned above that report reduced connectivity between certain brain areas, especially a reduced connectivity between STS and the core face processing regions in prosopagnosics (Avidan et al., 2013).

The tests’ reliability coefficients obtained for the controls and prosopagnosics reached good levels most of the time. However, for tests of holistic face processing employing static stimuli exclusively, in particular for the CFMT, prosopagnosics obtained only a fraction of the reliability coefficients of controls. We suggest that prosopagnosics use varying strategies (e.g., part-based processing) to compensate for their face recognition impairment, which leads to their inconsistent answering behavior in those tests. While we admit of course that this suggestion is a speculation from our part, the use of strategies by prosopagnosics has been widely reported in the literature (DeGutis, Bentin, Robertson, & D’Esposito, 2007; DeGutis, Cohan, & Nakayama, 2014). Future studies looking in more details at prosopagnosics’ responses in various tests should allow to uncover more evidence, and describe the consequences, of using strategies in tests of face processing.

That last finding, reduced test reliability when testing prosopagnosics, has important implications for our current study in particular and for research on prosopagnosia at large. An additional unsuccessful goal of our current study had been to assess a large group of prosopagnosics with a variety of tests with the goal of finding subgroups. In hindsight, after completion of our study, the general opinion is now that a much larger number of prosopagnosic participants is needed for finding clear subgroups, owing to various potential factors introducing noise in the test data, two of them being genetic diversity (Schmalzl et al., 2008) and co-morbidity (Mitchell, 2011). Our findings add a new factor to that list: reduced reliability in tests.

### Summary

With our extended battery of existing and newly created tests and our large sample size of prosopagnosic and control participants, we were able to refine our knowledge about face perception processes in general and for congenital prosopagnosia in particular. Furthermore, we are the first to reveal that the response behavior of prosopagnosics in tests for holistic processing differs from controls, as indicated by their noticeably reduced test reliability. Future work will need to examine the robustness and cause of this phenomenon. Additionally, better tests need to be designed, with higher reliabilities for prosopagnosics. Such tests would provide more robust results allowing to obtain a more accurate picture and better classification of the impairment.

## Notes

1. Please note the typo in Formula (2) for this reference. It should read as… {(k1–1)(n1–2))}…

2. Note, though, that in those studies only the partial design was used and only with upright faces.
